# Comparison of the Subgross Distribution of the Lesions in Invasive Ductal and Lobular Carcinomas of the Breast: A Large-Format Histology Study

**DOI:** 10.1155/2012/436141

**Published:** 2012-10-14

**Authors:** Syster Hofmeyer, Gyula Pekár, Mária Gere, Miklós Tarján, Dan Hellberg, Tibor Tot

**Affiliations:** ^1^Department of Pathology and Clinical Cytology, Central Hospital Falun, 79182 Falun, Sweden; ^2^Center for Clinical Research, and Department of Women's and Children's Health, Uppsala University, 75185 Uppsala, Sweden

## Abstract

To compare the lesion distribution and the extent of the disease in ductal and lobular carcinomas of the breast, we studied 586 ductal and 133 lobular consecutive cancers. All cases were documented on large-format histology slides. The invasive component of ductal carcinomas was unifocal in 63.3% (371/586), multifocal in 35.5% (208/586), and diffuse in 1.2% (7/586) of the cases. The corresponding figures in the lobular group were 27.8% (37/133), 45.9% (61/586), and 26.3% (35/133), respectively. When the distribution of the in situ and invasive component in the same tumors was combined to give an aggregate pattern, the ductal carcinomas were unifocal in 41.6% (244/586), multifocal in 31.6% (185/586), and diffuse in 26.8% (157/586) of the cases. The corresponding figures in the lobular category were 15.0% (20/133), 54.2% (72/133), and 30.8% (41/133), respectively. Ductal cancers were extensive in 45.7% (268/586), lobular in 65.4% (87/133) of the cases. All these differences were statistically highly significant (*P* < 0.0001). While the histological tumor type itself (ductal versus lobular) did not influence the lymph node status, multifocal and diffuse distribution of the lesions were associated with significantly increased risk of lymph node metastases in both ductal and lobular cancers.

## 1. Introduction

Breast cancer is a heterogeneous disease in which the individual cases deviate from each other in morphology, protein expression, molecular phenotype, genetic characteristics, and prognosis. Breast carcinomas of “special-types” have been delineated based on their microscopical characteristics, but the vast majority of tumors belongs to the category of not otherwise specified (NOS) ductal carcinomas. Invasive lobular carcinomas represent the most frequent “special-type” breast carcinoma and comprise 5–15% of all breast cancer cases [1]. In addition both the ductal and the lobular tumors also represent heterogeneous groups of diseases and can be prognostically stratified with grading or delineating distinct histological subtypes.

Numerous studies have compared ductal and lobular breast carcinomas using different criteria, and reported more [2, 3] or less favourable [4, 5] outcome in lobular compared to ductal carcinomas, or no significant differences in outcome [6, 7]. On the other hand, studies on subgross morphology (lesion distribution and disease extent) of these tumors are very rare. Tot has previously described the diffuse variant of invasive lobular carcinoma and reported a poorer prognosis when compared to unifocal and multifocal lobular cancers [8]. Foschini et al. [9, 10] studied in detail the subgross morphology of both in situ and invasive lobular carcinomas and observed that these tumors tended to be multifocal and extensive. A growing body of evidence exists regarding the biological and prognostic significance of tumor multifocality, diffuse lesion distribution, and extensive tumoral growth in breast cancer in general [11–13], but few studies have addressed this topic specifically in the ductal and lobular subgroups [14].

The present study was designed to compare the distribution of the in situ and invasive lesions and the extent of the disease in ductal and lobular carcinomas of the breast, and to evaluate the influence of these subgross morphological parameters on lymph node status in both histological categories.

## 2. Methods

### 2.1. Study Population

This study is a retrospective analysis of a consecutive series of breast carcinoma cases diagnosed at the Department of Pathology and Clinical Cytology of the County Hospital in Falun, Sweden, from January 2008 to July 2012. Patients with recurrent breast carcinomas that were initially diagnosed before the study period were excluded. In situ carcinomas with no invasive component, microinvasive (<1 mm) carcinomas, and patients who received preoperative neoadjuvant therapy were excluded. The remaining study population comprised 586 ductal, 133 lobular, and 102 invasive carcinomas of other histological types. Only the ductal and the lobular cancers were analyzed in the present study. The study was approved by the The Regional Ethical Review Board of Uppsala University.

### 2.2. Large-Section Histopathology

All specimens were prepared using the large-format histopathology method that has been performed routinely in our laboratory since 1982. The method has been described in detail elsewhere [15]. Briefly, all cases are discussed at a preoperative tumor board, and the radiological (mammography, ultrasound, and magnetic resonance imaging) appearance are recorded, including the radiological extent and distribution. This information, together with the whole-specimen radiograph received with the surgical specimen, guides the pathologist during the work up. The sector-resection specimens are sliced into 3-4 mm thick tissue slices parallel to the pectoralis fascia and are also radiographed. One to five of the most representative slices (measuring up to 9 × 8 cm) are selected and embedded in large paraffin blocks. Larger slices are bisected and embedded into separate blocks. Mastectomy specimens are sliced perpendicular to the pectoralis fascia to visualize the surgical margin in one histological level. All of the cases are further discussed at the postoperative tumor board to check the concordance of the radiological and histological findings. Most cases that are discrepant in favor of radiological findings can be resolved with additional specimen sampling for histological analysis.

## 3. Diagnostic Criteria

### 3.1. Histological Tumor Type

Invasive lobular carcinomas were defined by their cellular characteristics, growth pattern and E-cadherin expression, following the WHO criteria [1]. E-cadherin (DAKO, clone M3612) staining was performed routinely in all invasive carcinoma cases during the study period; the largest tumor focus was stained. Typical cases of invasive lobular cancer were built up of small uniform cells exhibiting a small, regular, darkly stained round, or oval nucleus. These cells often contained an intracytoplasmic vacuole of mucin pressing aside the small nucleus of the cells. The cells in invasive lobular carcinoma typically grew in cell files (one or two cell thick) or were haphazardly (diffusely) dispersed. Glandular lumina were absent in most cases. The two criteria, that is, typical cytological characteristics and typical histological growth pattern were used as alternative criteria, that is, tumors exhibiting both of these basic morphologic criteria (typical cells and typical histological growth pattern) were categorized as “classical” type of invasive lobular carcinoma, while tumors exhibiting only one of the criteria comprised the variants of it. This means that tumors consisting of typical cells were categorized as invasive lobular carcinomas irrespective of variations in histological growth patterns (tumors with solid, alveolar, and tubulolobular growth patterns were also included into lobular category). Tumors with typical histological growth pattern were also classified as lobular even if they consisted of different tumor cells. The vast majority of invasive lobular carcinomas exhibited complete loss of E-cadherin expression as demonstrated by immunohistochemistry. Cases with typical morphology and partial E-cadherin expression were, however, included into the lobular category. Cancers not showing any histological characteristics of special types tumors (including lobular cancers) in at least 90% of their cross-section surface were categorized as ductal carcinomas not otherwise specified. The vast majority of these tumors expressed E-cadherin.

The distributions of the invasive and the in situ components of the same lesion were determined separately using the previously published criteria by Tot [14]. The invasive component of the tumors was classified as follows: (1) unifocal tumors: one invasive focus observed in the large sections, with the tumor focus containing or not containing an in situ component. (2) Multifocal invasive lesions: multiple, well-delineated, invasive tumor foci separated from each other by uninvolved breast tissue, regardless of the distance between the foci. We did not analyze cases of “multicentricity” (defined as the presence of malignant structures in different quadrants of the same breast) separately, because it represents a clinical and/or radiological parameter; these cases were regarded as multifocal. (3) Diffuse tumors: tumors that are dispersed over a large area of the section, similar to a spider's web, with no distinct tumor mass. The in situ component of the tumors were regarded as “unifocal” if they seemed to involve a single terminal ductal lobular unit or several neighbouring terminal units without uninvolved breast tissue in between; they were regarded as “multifocal” if they involved several distant terminal ductal lobular units with uninvolved breast tissue in between, and as “diffuse” if they involved mainly the larger ducts. The distribution of the in situ and invasive components was combined with each other. Diffuse distribution of either in situ or invasive component qualified the lesion to be “diffuse.” Tumors without evidence of diffuse growth were classified as “multifocal” if either the in situ or the invasive component or both were multifocal.

**Figure 1:**
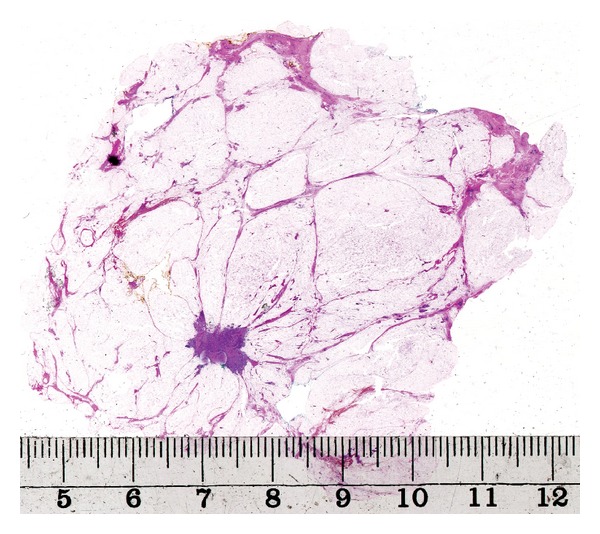
Large-format histology section showing a unifocal breast carcinoma.

**Figure 2:**
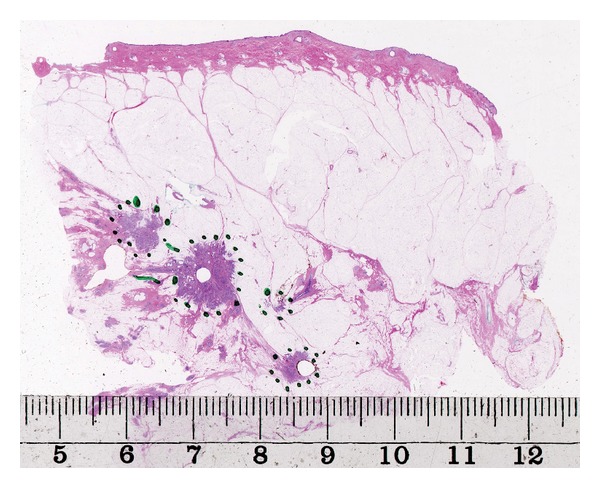
Large-format histology section showing a multifocal breast carcinoma.

**Figure 3:**
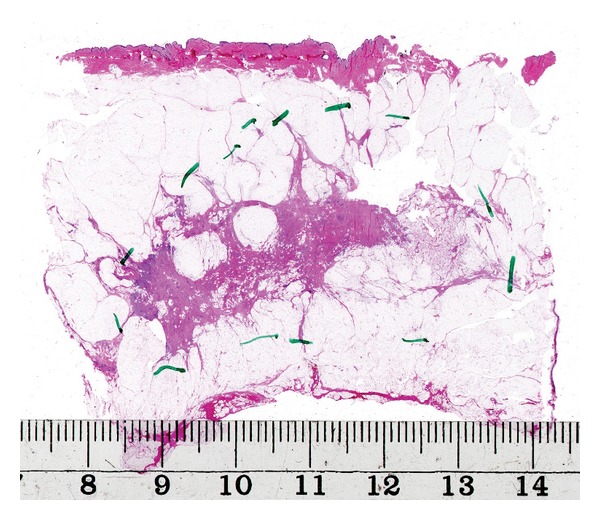
Large-format histology section showing a diffuse invasive carcinoma of the breast.

When the distribution of the lesions was assessed, an attempt was made in each case to summarize the findings in different tissue levels of the large sections to reconstruct the in vivo situation before surgery. Detailed correlations between radiological and pathological findings were essential. If a complete surgical intervention was performed in addition to the primary sector resection, an attempt was made to summarize the findings of the entire excised tissue. However, sector resection specimens (average size of 9 × 6 cm) were sufficient for categorizing the findings in most cases. Typical cases of unifocal, multifocal, and diffuse breast carcinomas are illustrated in Figures 1, 2 and 3.

Disease extent was defined as the tissue area in the large-format histology sections containing all the in situ and invasive malignant structures. Cases in which the tumor structures occupied an area 40 mm or larger in its largest dimension were categorised as extensive tumors [16] while the others were categorised as nonextensive.

### 3.2. Assessment of the Lymph Nodes

The axillary lymph nodes (both sentinel and nonsentinel nodes) were measured and sliced parallel to the longitudinal axis. Lymph nodes with a thickness of <5 mm were bisected, and thicker nodes were sliced to yield approximately 2 mm thick slices. Sentinel lymph nodes were examined during surgery with imprint cytology from all cut surfaces and frozen sections of 1-2 slices. The frozen sections were routinely stained and also stained intraoperatively with a cytokeratin 8/18 antibody (1 : 50, clone Cam 5.2; BD Biosciences). All slices were embedded in paraffin blocks; at least two sections from each of the blocks were stained with hematoxylin and eosin, and those from sentinel nodes were stained with the cytokeratin 8/18 antibody. Lymph node metastasis was assessed according to the sixth edition of the TNM staging system [17], which defines macrometastasis as at least one metastatic deposit >2 mm within a lymph node, micrometastasis as deposit(s) 0.2–2 mm, and isolated tumor cells as <0.2 mm deposits. For the purposes of the present study, cases with metastatic deposits 0.2 mm or greater in at least one of the examined lymph nodes were characterized as lymph node positive.

### 3.3. Study Execution

All of the large histological sections in this series and the slides from the lymph nodes were reviewed by two of the authors (TT, GP) for the purposes of the postoperative tumor board. Histological data, including the distribution of lesions, disease extent, tumor type, and lymph node status, were determined according to the diagnostic criteria described above and registered in a database. Statistical analyses (relative risk (RR) and comparison of proportions using the chi-square test) were carried out using commercially available software (MedCalc statistics for biomedical research; MedCalc Software, Belgium), with *P* values <0.05 regarded as significant.

## 4. Results

Among the 719 cases of the present series of newly diagnosed breast carcinomas, 586 were diagnosed as ductal and 133 as lobular applying the above-described criteria. The invasive component was unifocal in 408, multifocal in 269, and diffuse in 42 cases. Ductal carcinomas were unifocal in 63.3% (371/586), multifocal in 35.5% (208/586), and diffuse in 1.2% (7/586) of the cases. The corresponding figures in the lobular group were 27.8% (37/133), 45.9% (61/586), and 26.3% (35/133), respectively. These differences were highly significant indicating that the invasive component of lobular carcinomas is more often multifocal and much more often diffuse compared to ductal tumors (Table 1).

In situ component was not found in 5.3% (31/586) of the ductal and 5.2% (7/133) of the lobular cases. The in situ component of the other ductal tumors was unifocal in 42.8% (251/586), multifocal in 25.1% (147/586), and diffuse in 28.8% (157/586) of the cases. The corresponding figures in lobular tumors were 18.8% (25/133), 69.2% (92/133), and 6.8% (9/133), respectively. These differences were statistically also highly significant indicating that the invasive component of lobular carcinomas is more often multifocal compared to the ductal cases, and much less often diffuse.

When the distribution of the in situ and invasive components of the same tumors were combined, giving an aggregate pattern, the ductal carcinomas were unifocal in 41.6% (244/586), multifocal in 31.6% (185/586), and diffuse in 26.8% (157/586) of the cases. The corresponding figures in the lobular category were 15.0% (20/133), 54.2% (72/133), and 30.8% (41/133), respectively. These differences were also statistically significant, indicating that lobular carcinomas are more often multifocal than their ductal counterparts. The proportions of cases with diffuse combined lesion distribution were similar because of the frequent diffuse distribution of the in situ component in ductal carcinomas and the frequent diffuse distribution of the invasive component in lobular carcinomas.

The extent of the disease was assessable in all cases with exception of a single case of ductal and a single case of lobular cancer. Ductal cancers were extensive in 45.7% (268/586) of the cases compared to 65.4% (87/133) in lobular tumors (*P* < 0.0001).

There were no significant differences in the proportion of lymph nodes positive cases between ductal and lobular cancers in any lesion distribution category or in the extent categories. However, numerically, the proportions in almost all the categories were higher in ductal carcinomas than in lobular cases (Table 2).

In Table 3, the relative risk of having lymph node metastasis in ductal and lobular breast carcinomas by lesion distribution is given. Taking into account only the invasive component of the tumors in the entire study material, 26.7% (109/408) of the patients with unifocal tumors and 49.4% (133/269) of the patients with multifocal tumors had lymph node metastases giving a relative risk of 1.85 (95% CI 1.5136–2.2629, *P* < 0.0001). The relative risk of having lymph node metastasis in diffuse invasive tumors compared to unifocal ones was 2.05 (95% CI 1.4908–2.8184, *P* < 0.0001). In the group of ductal carcinomas, multifocality of the invasive component carried a relative risk of 1.96 (95% CI 1.5912–2.4106, *P* < 0.0001) of having lymph node metastasis compared to unifocal cases; the corresponding relative risk with diffuse distribution was 3.12 (95% CI 2.2088–4.4005, *P* < 0.0001). Multifocality of the invasive component of the lobular cases carried similar relative risk (RR = 1.82) which did not reach statistical significance (*P* = 0.1185). The relative risk (2.57) in diffuse versus unifocal invasive lobular carcinomas was statistically significant (*P* = 0.0136). As shown in Table 3, multifocality and diffuse distribution of the in situ component of the tumors carried lower relative risk of having lymph node metastases than multifocality or diffuse distribution of the invasive component in both the ductal and lobular categories. This was also observed regarding the aggregate (in situ + invasive components) lesion distribution.

## 5. Discussion

The proportions of multifocal breast cancer cases vary substantially in the literature, depending on definition, methodology of assessment, and criteria [18]. Most publications on this topic focused on the invasive component of the tumors, did not include the in situ component, and did not take diffuse distribution of the lesion into account. Tot published a system that included all components of the tumors and also the possibility of diffuse growth of both the in situ and invasive components. The criteria were tested on a consecutive series of 500 breast carcinoma cases documented on large-format histology sections [14] and are also described in detail in another report on the findings in 1000 consecutive cases in this special issue of the journal [19]. In the study published in 2007 [14], 34% (170 of 500 cases) of the cases were unifocal, 36% (180 of 500 cases) were multifocal, and 28% (138 of 500 cases) were diffuse when the in situ and invasive components of the tumor were combined into an aggregate pattern. These results are almost identical to the results in the study on 1000 cases [19] in which 36% (366/1000) unifocal, 35% (347/1000) multifocal and 28% (280/1000) diffuse cases were found. The corresponding figures in the present study are very similar (36.7% unifocal, 35.8% multifocal, and 27.5%, diffuse tumor growth). In our previous studies, we found about 45% of the breast cancer cases being extensive (occupying a tissue are ≥40 mm in the largest dimension) which was confirmed in the present series [16].

Foschini et al. studied the distribution of the lesions (both in situ and invasive) in lobular carcinomas of the breast through three-dimensional stereomicroscopy of thick large-format histological sections in fifteen cases and demonstrated multiple tumor foci in most cases [9]. The in situ component was multicentric in nine of their cases and the average maximum distance among the in situ foci was 37.9 mm, while the average maximum distance among the invasive foci was 58.2 mm. Although this study was performed on a limited number of cases, the results clearly indicated frequent multifocality of the lesions and extensive tumoral growth in lobular carcinomas.

In another seminal paper by Foschini and coworkers [10], the advantages of large-format histology were used to assess the distribution of the lesions in in situ and invasive ductal breast carcinomas. They found that the in situ component was multifocal in 42 of their 45 cases, but the invasive component was multifocal in only four of their cases. They also frequently found low-grade carcinoma in situ to be widely spread in the breast.

In 2003, Tot described the diffuse variant of the invasive lobular carcinoma [8] and reported the distribution of the lesions in 130 consecutive cases of invasive lobular carcinomas diagnosed at the Department of Pathology in Falun, Sweden during the period 1991–1997. All these tumors were documented on large-format histological sections and the patients were followed up for on average 78 months (range 4–131 months). In that study, 39% of the cases were unifocal, 12% multifocal, 28% diffuse while 19% were combined (the term “combined” designated those cases which contained only minor areas of diffuse growth of the tumor cells and being otherwise multifocal). The distribution of the in situ component was not analysed in that series of cases. In the present paper we report novel results on a series of consecutive cases diagnosed at the same department during the period Jan 2008–Jul 2012 and worked up with the same method of large-format histology. Compared to the previous study, the design was extended to include also analyses of the in situ component, not only in lobular but also in ductal tumors, and analyzing the extent of the disease and the lymph-node status. In the present study we avoided the “combined” category of the distribution of the invasive component and classified the cases based on the dominant pattern of invasion. This explains the differences in the proportions of unifocal and multifocal cases between these two studies.

Our present study showed that there are statistically highly significant differences in lesion distribution between invasive lobular and invasive ductal carcinomas in all analyzed scenarios (only invasive component, only in situ component, and in situ and invasive components combined). The differences clearly indicate that the invasive component of lobular carcinomas compared to ductal carcinoma tends to be multifocal more often (45.9% versus 35.5%) and much more often diffuse (26.3% versus 1.2%). On the contrary, the in situ component of the ductal carcinomas as compared to lobular carcinoma was much more often diffuse (28.8% versus 6.8%) and much less often multifocal (25.1% versus 69.2%). This also resulted in significant differences in combined lesion distribution between ductal and lobular carcinomas. We also observed that lobular carcinomas were significantly more frequently extensive than their ductal counterparts.

The majority of the publications related to metastatic potential of multifocal breast carcinomas reported higher risk of lymph node involvement in multifocal than in unifocal cases [20–23]. In our recently published series [23], 25.6% of the unifocal, 53.4% of the multifocal and 35.7% of the diffuse cases had lymph node metastases (tumor deposits >0.2 mm). In another a previous study, we found that the odds ratio of having lymph node metastasis was 2.33 for diffuse carcinomas compared to unifocal tumors [14]. Similar results were generated in the present study. Although the differences between the proportions of the lymph node positive cases were significant between unifocal, multifocal and diffuse cases, no such differences could be related to the histological tumor type (ductal versus lobular) in the present series.

The present study reported several histopathological significant differences between ductal and lobular tumors. In conclusion, the invasive component of the lobular carcinomas was more often multifocal and diffuse than the invasive component of the ductal tumors. The in situ component was more often multifocal but less often diffuse in lobular carcinomas. Lobular carcinomas were more often extensive than the ductal tumors. The histological tumor type (ductal versus lobular) was not directly related to the metastatic potential of the tumors, but multifocal and diffuse lesion distribution (especially that of the invasive component) significantly increased the relative risk of having lymph node metastasis in both ductal and lobular breast carcinomas.

## Figures and Tables

**Table 1:** Lesion distribution and disease extent in ductal and lobular breast carcinomas, Dalarna, Jan 2008–Jul 2012.

			Ductal	Lobular	*χ* ^2^ test
	Invasive component	Unifocal	63.3% (371/586)	27.8% (37/133)	*P* < 0.0001
		Multifocal	35.5% (208/586)	45.9% (61/586)	
		Diffuse	1.2% (7/586)	26.3% (35/133)	
Lesion distribution	In situ component*	Unifocal	42.8% (251/586)	18.8% (25/133)	*P* < 0.0001
		Multifocal	25.1% (147/586)	69.2% (92/133)	
		Diffuse	28.8% (157/586)	6.8% (9/133)	
	Combined in situ + invasive components	Unifocal	41.6% (244/586)	15.0% (20/133)	*P* < 0.0001
		Multifocal	31.6% (185/586)	54.2% (72/133)	
		Diffuse	26.8% (157/586)	30.8% (41/133)	

Disease extent**	Nonextensive		54.1% (317/586)	33.8% (45/133)	*P* < 0.0001
	Extensive		45.7% (268/586)	65.4% (87/133)	

Total			586	133	719

^∗^5.3% (31/586) of the ductal and 5.2% (7/133) of the lobular cases had no demonstrable in situ component.

^∗∗^The extent of the disease was not assessable in 0.2% (1/586) of the ductal and 0.8% (1/133) of the lobular cancers.

**Table 2:** Proportion of cases with lymph node metastases in ductal and lobular carcinomas by lesion distribution and disease extent, Dalarna, Jan 2008–Jul 2012.

			Ductal	Lobular	Comparison of proportions
	Invasive component	Unifocal	27.5% (102/371)	18.9% (7/37)	*P* = 0.9375 (−35.3%–29.4%)
		Multifocal	53.8% (112/208)	34.4% (21/61)	*P* = 0.1486 (−5.5%–41.2%)
		Diffuse	85.7% (6/7)	48.6% (17/35)	*P* = 0.2713 (−16.7%–65.1%)
Lesion distribution	In situ* component	Unifocal	33.1% (83/251)	16.0% (4/25)	*P* = 0.8706 (−42.2%–36.5%)
		Multifocal	43.5% (64/147)	40.2% (37/92)	*P* = 0.8549 (−17.3%–24.4%)
		Diffuse	43.3% (68/157)	33.3% (3/9)	*P* = 0.7997 (−48.7%–44.6%)
	Combined in situ + invasive components	Unifocal	29.9% (73/244)	20.0% (4/20)	*P* = 0.8918 (−48.1%–33.1%)
		Multifocal	42.2% (78/185)	31.9% (23/72)	*P* = 0.5360 (−15.1%–31.2%)
		Diffuse	43.9% (69/157)	43.9% (18/41)	*P* = 0.7898 (−26.0%–27.6%)

Disease extent**	Nonextensive		27.1% (86/317)	26.7% (12/45)	*P* = 0.7285 (−23.1%–33.4%)
	Extensive		50.0% (134/268)	37.9% (33/87)	*P* = 0.2975 (−8.5%–30.5%)

Total			586	133	

^∗^5.3% (31/586) of the ductal and 5.2% (7/133) of the lobular cases had no demonstrable in situ component.

**The extent of the disease was not assessable in 0.2% (1/586) of the ductal and 0.8% (1/133) of the lobular cancers.

**Table 3:** Relative risk of having lymph node metastasis in ductal and lobular breast carcinomas by lesion distribution, Dalarna, Jan 2008–Jul 2012.

		Unifocal	Multifocal	Diffuse	Relative risk multifocal versus unifocal	Relative risk diffuse versus unifocal
	Ductal	27.5% (102/371)	53.8% (112/208)	86.7% (6/7)	RR = 1.9585 *P* < 0.0001 (1.5912–2.4106)	RR = 3.1176 *P* < 0.0001 (2.2088–4.4005)
Invasive component	Lobular	18.9% (7/37)	34.4% (21/61)	48.6% (17/35)	RR = 1.8197 *P* = 0.1185 (0.8582–3.8585)	RR = 2.5673 *P* = 0.0136 (1.2138–5.4303)
	Total	26.7% (109/408)	49.4% (133/269)	54.8% (23/42)	RR = 1.8507 *P* < 0.0001 (1.5136–2.2629)	RR = 2.0498 *P* < 0.0001 (1.4908–2.8184)

	Ductal	33.1% (83/251)	43.5% (64/147)	43.3% (68/157)	RR = 1.3534 *P* = 0.0192 (1.0506–1.7435)	RR = 1.3098 *P* = 0.0351 (1.109–1.6835)
In situ* component	Lobular	16.0% (4/25)	40.2% (37/92)	33.3% (3/9)	RR = 2.5153 *P* = 0.0526 (0.0989–6.3841)	RR = 2.0833 *P* = 0.2642 (0.5743–7.5575)
	Total	31.5% (87/276)	42.3% (101/239)	42.8% (71/166)	RR = 1.3406 *P* = 0.0119 (1.0668–1.6848)	RR = 1.3569 *P* = 0.0156 (1.0595–1.7377)

	Ductal	29.9% (73/244)	42.2% (78/185)	43.9% (69/157)	RR = 1.4093 *P* = 0.0085 (1.0913–1.8198)	RR = 1.4690 *P* = 0.0039 (1.1316–1.9069)
Combined in situ + invasive components	Lobular	20.0% (4/20)	31.9% (23/72)	43.9% (18/41)	RR = 1.5972 *P* = 0.3284 (0.6245–4.0854)	RR = 2.1951 *P* = 0.1020 (0.8555–5.6328)
	Total	29.2% (77/264)	39.3% (101/257)	43.9% (87/198)	RR = 1.3474 *P* = 0.0156 (1.0581–1.7158)	RR = 1.5065 *P* = 0.0011 (1.1790–1.9250)

*5.3% (31/586) of the ductal and 5.2% (7/133) of the lobular cases had no demonstrable in situ component.
